# From principles to practice: Implementation of entrustable professional activities (EPAs) for surgical pathology residency education in a large academic hospital

**DOI:** 10.1016/j.acpath.2023.100097

**Published:** 2023-11-20

**Authors:** Christopher Felicelli, Alcino Gama, Yevgen Chornenkyy, Bonnie Choy, Luis Z. Blanco, Jorge E. Novo

**Affiliations:** Department of Pathology, Northwestern University Feinberg School of Medicine, Chicago, IL, USA

**Keywords:** Competency-based medical education, Entrustable professional activities, Graduated responsibility, Medical education, Surgical pathology

## Abstract

Over the past decade, competency-based medical education (CBME) has gained momentum in the United States to develop trainees into independent and confident physicians by the end of their training. Entrustable professional activities (EPAs) are an established methodology for assessing trainee development through an outcomes-driven rather than a time-based model. While EPAs have been utilized as an assessment tool for CBME in Europe and Canada, their validation and implementation in some medical specialties has occurred more recently in the United States. Pediatrics was the first specialty in the US to conduct a large-scale UME-GME pilot. Pathology Residency EPAs were published in 2018; however, implementation in training programs has been slow. We have piloted EPAs in our residency program's surgical pathology rotation and propose a unique set of 4 surgical pathology EPAs to track trainee preparedness for independent practice.

## Introduction

Entrustable professional activities (EPAs) were first described in the Netherlands in the early 2000s as a way to reshape traditional education models from a time-based model to an outcome-driven model.^1^^,^^2^ EPAs can serve as an essential clinical assessment component of the competency-based medical education (CBME) model and offer the opportunity to operationalize competency evaluation and related entrustment decisions in the course of regular patient care, and address some of the challenges educators and trainees have faced in bridging core competency theory into clinical practice and performance assessment. Traditionally, undergraduate and graduate medical education in the United States has followed a regimented, linear learning model in which curriculum, expectations, and performance are determined in a fixed period based on the training year; however, the utilization of EPAs has more recently been implemented, with specialties such as surgery transitioning to EPAs for the evaluation of surgery residents in July 2023.^3^, ^4^, ^5^, ^6^ As initially described in Dr. Ten Cate's original framework of EPA creation,^2^ the specialty-specific EPAs should incorporate unique training qualities, in-the-moment decisions, and measurements that would reflect the natural progression from direct supervision to entrusted unsupervised practice for patient care. Currently, pathology resident and fellow assessment occurs biannually and is based on the Accreditation Council for Graduate Medical Education (ACGME) milestones, a uniform assessment method in 6 key general competencies across anatomic and clinical pathology.^7^ While these evaluations provide a uniform assessment tool, having the ability to tailor expectations and performance assessments to an individual resident can be a challenging task.

CBME has recently been a focus of ACGME and the American Board of Medical Specialties (ABMS).^8^^,^^9^ Efforts are underway in the US to implement CBME and develop specialty-specific EPAs for graduate medical education (GME) and undergraduate medical education (UME) in surgery, pediatrics, internal medicine, radiology, and family medicine.^10^, ^11^, ^12^, ^13^ In February 2022, the American Board of Surgery (ABS) announced the move to a competency-based assessment of surgical trainees by introducing the ABS-EPA Project, launching in July 2023 for general surgery residency programs.^14^ The American Board of Pediatrics (ABP) plans to integrate EPA data into certification decision-making by 2028. Both ABP and ABS are aligning their EPAs with the ACGME/ABMS core competencies and ACGME milestones as part of a unified approach to CBME and assessment. Several studies have validated both ABS's and ABP's EPAs as reliable tools for assessing levels of entrustment in residents.

In 2017, the Graduate Medical Education Committee of the College of American Pathologists undertook a multiyear endeavor to develop EPAs specific to the field of pathology.^15^ From this group, 19 unique EPAs were created, spanning the depth of Anatomic and Clinical Pathology's depth to reflect the ACGME milestones' goals. Although these EPAs were novel for pathology, they are general in their scope, with limited implementation thus far. A multi-institutional pilot addressing four of these EPAs was completed, and publication is pending.^16^ Additionally, Hematopathology published EPA guidelines for fellowship training.^17^ However, implementation of CBME and EPAs in pathology residency and fellowship programs is limited at this time. Current barriers include the timing of implementation, the identification of “EPA Champions” (i.e., willing faculty prepared for EPA assessment and utilization), and the continued utilization of EPAs in assessment. The American Board of Pathology (ABPath) has been collecting information regarding the opinion of “new in practice”: diplomates on various aspects of residency and selected subspecialty fellowship training since 2014 and found graduated responsibility to be an area that needs to be improved in training programs. Other notable areas for improvement included lab administration, molecular pathology, and clinical informatics.^18^^,^^19^ Additionally, in 2020, ABPath's Board of Trustees approved the support of a Promotion in Place (PIP) pilot, which tests the use of CBME across the specialties at Massachusetts General Hospital. ABPath is investigating additional options to further competency-based assessment in training and certification.

Using the Royal College of Physicians and Surgeons of Canada's (RCPSC) Pathology EPA Guide as a framework, we aimed to develop pathology EPAs specific to our anatomic pathology (AP) program's surgical pathology rotation.^20^ These incorporated ACGME milestones are measurable and comprehensive for both trainee and the evaluator. To our knowledge, we are the first United States-based pathology residency program to develop detailed EPAs specific to surgical pathology training.

## Material and methods

A holistic review of our current surgical pathology rotation evaluation assessments was performed over one year. As a result, deficiencies in evaluation methodology, including graduated responsibility shortcomings, were identified. The review process was performed by the director of the surgical pathology rotation in a large academic hospital, with assistance from senior pathologists with specializations in education and faculty development.

A comprehensive literature search was performed to evaluate the history, feasibility, value, and scalability of the creation and implementation of EPAs in pathology residency training. In addition, previous models developed by the CAP Graduate Education Committee (GME) and the RCPSC were reviewed to identify potential training similarities and differences concerning EPA development.

Following a pilot implementation of the EPA forms, a 6-month assessment survey was offered to 12 faculty and 22 residents. Both surveys targeted the ease, understanding, feasibility, and impact of EPA implementation in multiple. Each survey had 8 questions, and the answers were graded from 1 (strongly disagree) to 5 (strongly agree).

Following the preliminary implemental period, the data from EPA forms were aggregated and compared across surgical pathology training timelines to assess education and development.

## Results

**Fig. 1:**
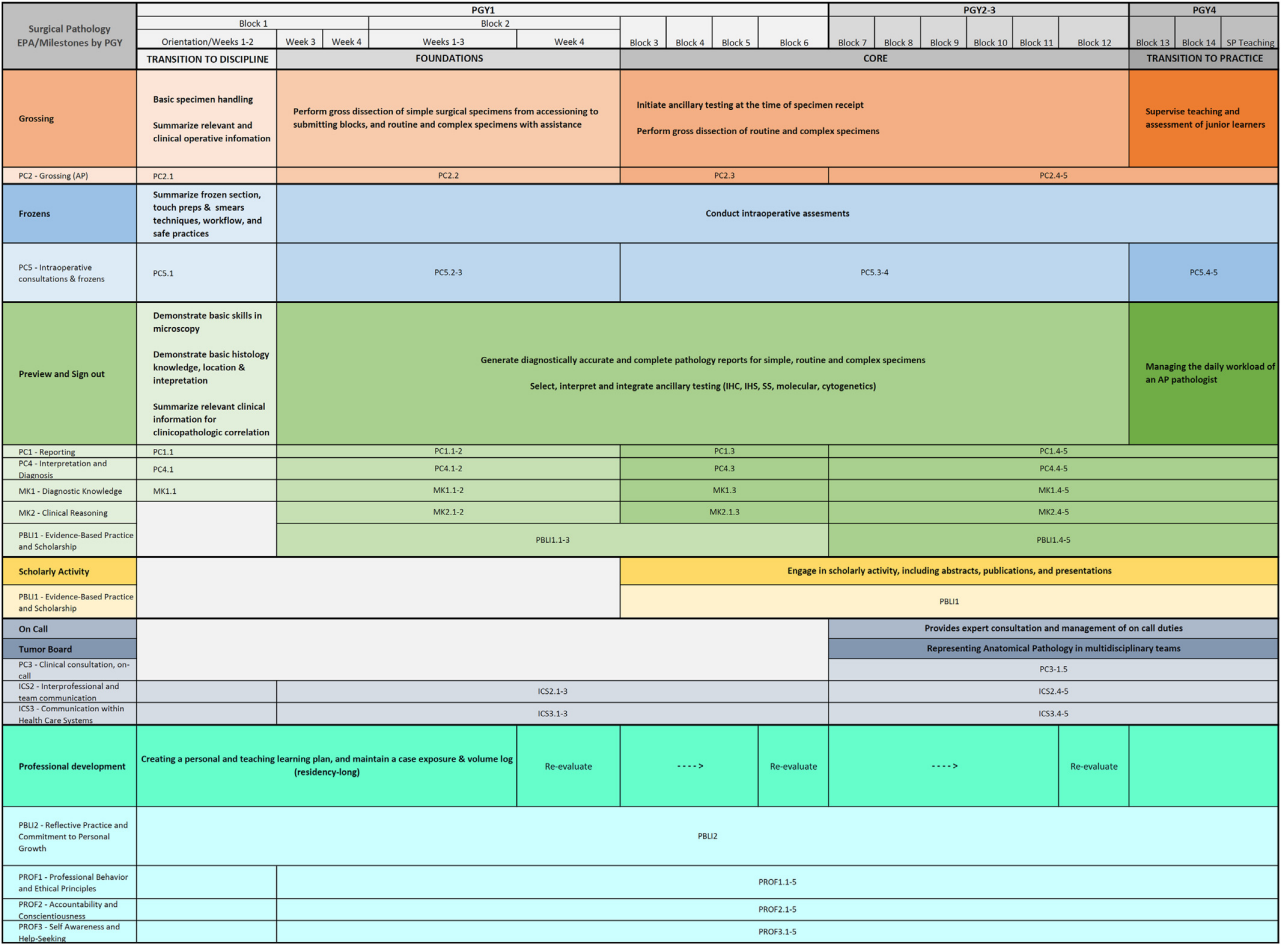
Surgical pathology rotation model with EPA and milestone breakdowns.

We identified 4 EPAs for surgical pathology training, Grossing, Frozens, Preview/Sign-out, and Professional Development, each broken down into four overarching categories based on the Canadian Model, focused on outcome-based measures instead of a time-based assessment. (Fig. 1). The following outcomes-based categories were as follows: Transition to Discipline, Foundations, Core, and Transition to Practice. Within each EPA, knowledge and individual skills for development within each outcomes-based measure are provided to assess and track resident progression. (Table 1).

**Table 1:** Entrustable professional activities and key competencies for surgical pathology (after the Canadian RCPSC Competency by Design Model of CBME).

EPA	Transition to discipline	Foundations	Core	Transition to practice
Grossing	Participation in Basic specimen handling Summarizing relevant clinical information for clinicopathologic correlation Demonstrating basic skills in microscopy	Performing gross dissection of simple surgical specimens from accessioning to submission of blocks	Performing gross dissection of routing surgical specimens Performing gross dissection of complex surgical specimens	Supervising, teaching, and assessing junior learners
Frozens	Summarize frozen section, touch prep and smears technique, workflow, and safe practices in intraoperative consultations		Conducting intraoperative assessments	
Preview and Sign-out	Demonstrate basic skills in microscopy Demonstrate basic histology knowledge, tissue location, and interpretation	Microscopic reviewing and reporting of simple surgical specimens	Initiating ancillary studies at the time of specimen receipt Generating diagnostically accurate and complete pathology reports for simple, routine, and complex surgical pathology cases Selecting, interpreting, and integrating ancillary diagnostic techniques	Managing the daily workload of an anatomical pathologist including surgical pathology, intraoperative consultations, and outside consult cases
Professional Development	Creating a personal and teaching learning plan, and maintain a case exposure and volume log		Representing anatomical pathology in multidisciplinary teams Maintaining a personal learning and career plan	Developing and implementing a personal learning plan geared to setting of future practice

The content provided in each EPA is as follows: (1) an EPA category, (2) an EPA title, (3) a summary of the EPA, (4) an assessment and plan, (5) the relevant ACGME milestones, and (6) an evaluation type. Evaluation forms provide the resident with actionable, semi-quantifiable feedback to improve and progress to the next EPA category. The entire set of reference materials for each EPA, the outcomes-based categories, the assessed knowledge and skills, and all evaluation forms are available within the Supplemental Data S1.

We used the Canadian CBME model in which “Transition to Discipline” encompasses what is expected from a new Pathology trainee.^20^ This period reflects what is expected in an orientation period, incorporates level 1 of the ACGME milestones, and has a goal completion of 2 weeks (Table 2). The basics of grossing, intraoperative consultations, preparation of case sign-out, and beginnings of professional development comprise the basis of the Transition to Discipline. Within the grossing category of the Transition to Discipline, residents participate in basic specimen handling and summarize relevant clinical information for clinicopathologic correlations. The frozen category will employ residents to summarize frozen sections, touch preparation, and smear techniques while understanding workflow and safe practices. Preparation of case sign-out entails demonstrating basic skills in microscopy and basic histology. At this point, residents will create a personal learning plan while maintaining a case exposure and volume log. ACGME milestones assessed pertain to the first level of PC1, PC2, PC4, PC5, MK1, and PBLI2.

**Table 2:** Transition to discipline, sample grossing EPA.

Participating in basic specimen handling	Assessment plan:	Evaluation type:
Use of the basic knowledge covered in the orientation to the laboratory in order to:	Part A: Specimen handling	Direct observation of specimen type by teaching resident or PA, minimum 5 observations.
	Direct observation or case review by teaching/senior resident or PA.	
• Match requisition and container and/or specimen		Final written exam or oral exam after surg path block in 1st year (ME 1.3)
• Systematically verify the adequacy of patient and clinical information (requisition adequacy and completeness such as documentation of ischemic time) to initiate laboratory evaluation of a specimen	Use form 1A^a^	Clinical scenario:
• Select and recognize the appropriate fixative type (formalin, alcohol) and assess whether the quantity and size of the specimen container is appropriate		
• Match slides, blocks, and requisition	Collect 5 observations of achievement per subspecialty bench	
	Part B: Assessment of knowledge	
	Evidence of satisfactory completion of a structured oral or written quiz administered by the supervising pathologist	
	Collect 1 observation of achievement	
	Relevant milestones:	
**Summarizing relevant clinical information for clinicopathologic correlation**	**Assessment Plan:**	**Evaluation type:**
Extracting clinical information, including clinical history, relevant laboratory and imaging results from a number of different sources (including electronic), interpreting this information in light of the clinical question, and providing a summary.	Case discussion and/or review of written clinical summary by supervisor (PA, teaching resident, or attending pathologist)	Observations/case discussion by pathologist while resident presents a case (after synthesizing relevant info from various sources) before sign out.
	Use Form 1A^a^	
	Collect 5 observations of achievement per subspecialty bench	
	**Relevant milestones:**	
	PC2.1	
**Demonstrating basic skills in microscopy**	**Assessment Plan:**	**Evaluation type:**
Using a microscope correctly and troubleshooting its principal problems	**Part A: Microscopy**	Direct observation by supervisor
	Direct observation by supervisor.	Structured oral or written quiz administered by the supervising pathologist
This includes:	Collect at least 1 observation of achievement	Clinical scenarios:
• Setting up the microscope (turning it on, adjusting the focus), using a polarizer and micrometer, understanding ergonomic setup	**Part B: Assessment of knowledge**	Orientation, preview, sign out
• Performing basic microscope maintenance such as changing objectives and bulbs	Evidence of satisfactory completion of a structured oral or written quiz administered by the supervising pathologist	
• Viewing the slide	Collect 1 observation of achievement	Apply knowledge of how a light microscope works
	**Relevant milestones:**	- Perform basic microscope maintenance
	PC1.1	- Direct observation on day 1 by a coresident/staff of adjusting a microscope to create an image.
	PC4.1	
	MK1.1	
**Demonstrate basic histology knowledge, tissue location, and interpretation**	**Assessment Plan:**	**Evaluation type:**
Demonstrating basic knowledge of normal histologic	**Part A: Interactive session**	Demonstration by supervisor
	Slide session provided by pathology faculty, with review of normal histology, patterns, and cellular details.	Structured oral or written quiz administered by the supervising pathologist
This includes:	**Part B: Assessment of knowledge**	
• Knowledge of normal histology of the most common organs.	Evidence of satisfactory completion of a structured oral or written quiz administered by the supervising pathologist	Clinical scenarios:
• Be able to describe histologic features at low power (pattern, borders) and high power (nucleus, chromatic, cytoplasm, cell shape)	Collect 1 observation of achievement	Orientation
• Utilize basic knowledge to identify tissue sources from unknown location.	**Relevant milestones:**	
	PC4.1	
	MK1.1	
**Summarize frozen section, touch prep and smears technique, workflow, and safe practices in intraoperative consultations**	**Assessment plan:**	**Evaluation type:**
Demonstrating practical knowledge and familiarity of the intraoperative consultation workflow	**Part A: Orientation session**	Demonstration by supervisor, direct observation practicing technique in gross room
	Orientation session provided by pathology faculty, with overview of the intraoperative consultation workflow, and demonstrating safe technique for performing frozen sections, touch preps, and smears	
This includes:		Clinical scenarios:
• Step by step knowledge on performing a frozen section, a touch prep, and a smear.	**Part B: Assessment of knowledge**	Orientation, gross room
• Be familiar with the location of tools, and equipment.	Evidence of satisfactory preparation of a frozen section, a touch prep, and a smear by direct observation from the supervising pathologist	
• Demonstrate safe technique.	**Relevant milestones:**	
	PC5.1	
**Creating a personal and teaching learning plan, and maintain a case exposure & volume log**	**Assessment Plan:**	**Evaluation type**
• Creating and maintaining a clinical training portfolio	**Part A: Clinical training portfolio**	This would be direct observation of the case log, ensuring adequacy of information storage, and demonstration of a learning plan, identifying self-deficiencies, learning sources, and a plan for evaluation
• Presenting a personal learning project, including identifying a topic, and identifying and utilizing information sources, (including self-assessment).	Resident's submission of teaching and learning plan reviewed by surgical pathology rotation director, residency program director, or assistant residency program director	Clinical Scenario:
		End of rotation
	Use form 1B^a^	Specific rotation:
	Collect 1 observation of achievement	Performed by rotation director at end of four specific blocks: orientation, block 2, block 6, block 12
	**Part B: Personal learning project**	
	Direct observation by rotation director	
	Use form 4^a^	
	Collect 1 observation of achievement	
	**Relevant milestones:**	
	PBLI2	

a Forms 1A, 1B, and 4 are available as Supplemental Data S1.

The Transition to Discipline additionally has forms to track resident progression and provide semi-quantitative data to assess development. Modified from Gomes et al.,^4^ an intraoperative consultation evaluation form was created to measure resident development independently by performing frozen section analysis (Supplemental Data S1, Form 2). The assessment form is broken down into 11 distinct categories, 8 of which are scored on a scale of 1–5, with 1 representing “I had to do it” (i.e., the attending pathologist having to perform all parts of the frozen) and 5 representing “I did not need to be there” (i.e., the resident is independent enough to perform the specific aspect with oversight only). Case preparation, history gathering, technical performance, and diagnostic interpretations are all aspects assessed within the form. Additional assessment forms in the Transition to Discipline include grossing basics and the development of a learning plan (Supplemental Data S1).

The “Foundations” category comprises the next set period of transition in a resident's development in surgical pathology. The ideal timeframe for Foundations is for the resident's first two blocks (4 weeks/block) on surgical pathology, where junior residents begin independent grossing and previewing without a senior resident. “Foundations” is built upon the framework of Transition to Discipline, further emphasizing the grossing and preparation of case sign-out categories. Within “Foundations,” residents will now perform gross dissection of simple surgical specimens (for example, non-neoplastic thyroids, leiomyoma uteri, and prophylactic mastectomies). Additionally, there is more emphasis on preparation for case sign-out, with independent case previewing without a senior resident and beginning to learn how to report simple surgical specimens. ACGME milestone levels 1–2 are assessed at this time. Evaluation forms to assess sign-out preparedness, efficiency, and workflow are also implemented as another direct, actionable way to measure resident progression (Supplemental Data S1, Form 3).

After proper assessment and completion of the “Foundations” category, the resident progresses to the “Core” category. This category comprises the majority of the resident's training while in surgical pathology for the next three years. In the “Core” category, the Grossing EPA involves the macroscopic examination and sampling of routine and complex surgical specimens. Preparation of case sign-out now involves independently initiating ancillary testing (molecular, cytogenetics, IHC), generating diagnostically accurate and complete pathology reports, and selecting, interpreting, and integrating ancillary diagnostic techniques. In addition, the resident will lead frozens and independently conduct intraoperative consultations. For professional development, residents will begin to represent the department at multidisciplinary teams (tumor boards) and continue to maintain personal learning and career plans. Levels 3–5 of ACGME milestones are utilized at this time. All prior evaluation forms are continuously utilized to continue longitudinal development tracking and provide the resident with immediate actionable feedback.

The final category of surgical pathology development is the “Transition to Practice,” which in the Canadian model occurs in the 5th year of training. Residents should reach this level by their fourth year of training when they step into a trainer and pre-fellow role. The grossing EPA is supplemented by supervising, teaching, and assessing junior residents. Preparation for case sign-out now encompasses managing the entire daily workload of an anatomic pathologic, including all in-house cases, intraoperative consultations, and outside cases. Professional development should be solidified by developing personal learning plans geared to the setting of future practice. Residents ideally should reach levels 4–5 of the ACGME milestones by the end of their Transition to Practice.

**Fig. 2:**
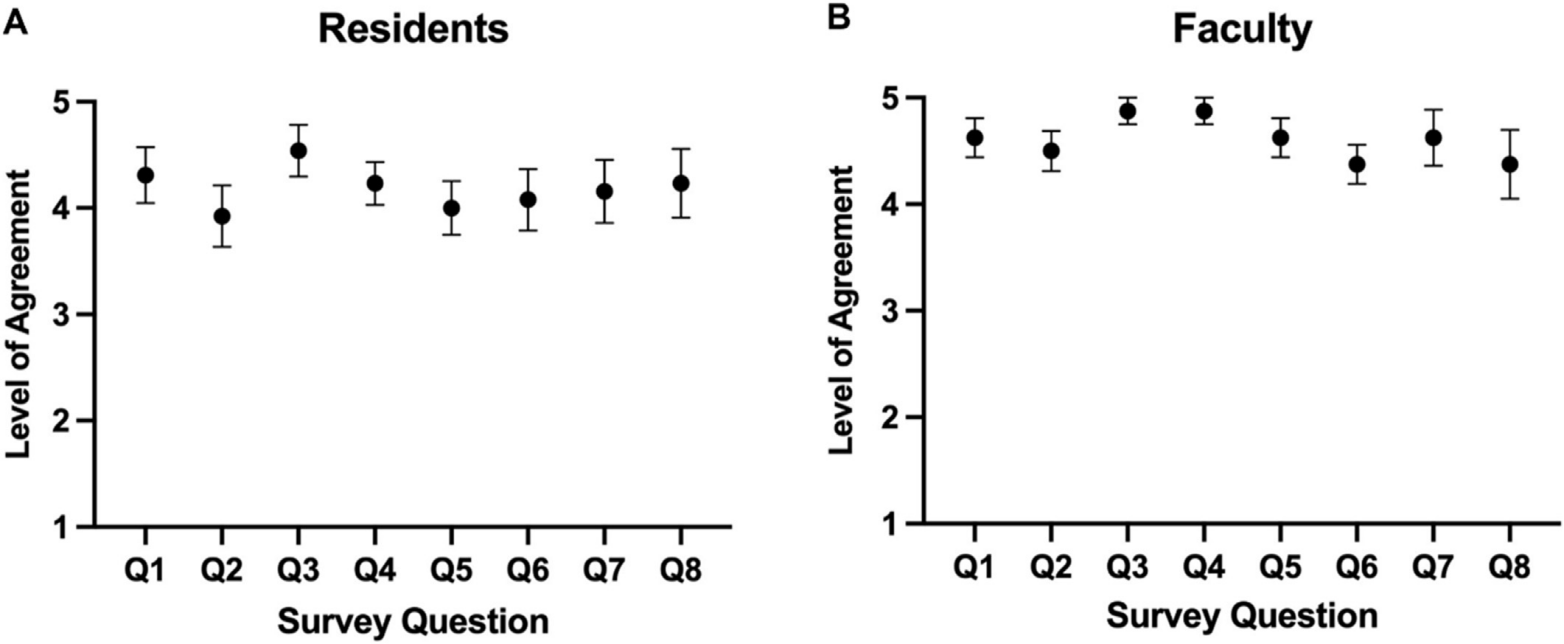
Survey responses for ease of use, feasibility, and impact of the EPA forms for residents (**A**) and Faculty (**B**). Legend: 1-strongly disagree, 2-disagree, 3-neutral, 4-agree, 5-strongly agree.

Following the implementation of the EPA evaluation forms, a 6-month assessment survey with 8 questions was offered to residents (Table 3) and faculty members (Table 4). The Qualtrics survey received responses from 13 pathology trainees (59%) and 8 faculty members (67%). The trainee responses are summarized in Fig. 2A, which shows that overall, trainees agreed with the survey questions regarding the ease and feasibility of using EPAs). Notably, trainees responded with high concordance that the EPAs and their associated evaluations set good benchmarks for training (4.54 ± 0.24), are easy to understand (4.30 ± 0.26), are easy to use (4.23 ± 0.20), and should continue to be used (4.23 ± 0.20). Similarly, the faculty responses are summarized in Fig. 2B, which shows that, overall, faculty also agreed with the survey questions regarding the ease and feasibility of using EPAs, with trends towards solid agreement. Notably, faculty agreed highly that EPAs and their associated evaluations set good benchmarks for training (4.88 ± 0.13), are easy to use (4.88 ± 0.13), do not consume a significant amount of time (4.63 ± 0.19), and accurately reflect the trainee's performance (4.63 ± 0.26).

**Table 3:** Survey questions assessed about ease, feasibility, and impact of EPAs on residents.

Q1	The EPA forms and scale and easy to understand.
Q2	Each individual parameter explanation is easy to understand.
Q3	The parameters being measured are important benchmarks for successful practice in pathology.
Q4	The EPA forms are easy to use.
Q5	The EPA forms accurately reflect my performance.
Q6	The EPA forms help me focus on specific skills to improve.
Q7	The EPA forms help me receive better feedback on my performance.
Q8	EPA forms should be used by all faculty and continue to be utilized.

**Table 4:** Survey questions assessed about ease, feasibility, and impact of EPAs on faculty.

Q1	The EPA forms and scale and easy to understand.
Q2	Each individual parameter explanation is easy to understand.
Q3	The parameters being measured are important benchmarks for successful practice in pathology.
Q4	The EPA forms are easy to use.
Q5	The EPA forms DO NOT consume a significant amount of time compared to informal (non-standardized) feedback.
Q6	The EPA forms allow me to give better feedback to the trainee.
Q7	The EPA forms accurately reflect the trainee's performance.
Q8	EPA forms should be used by all faculty and continue to be utilized.

**Fig. 3:**
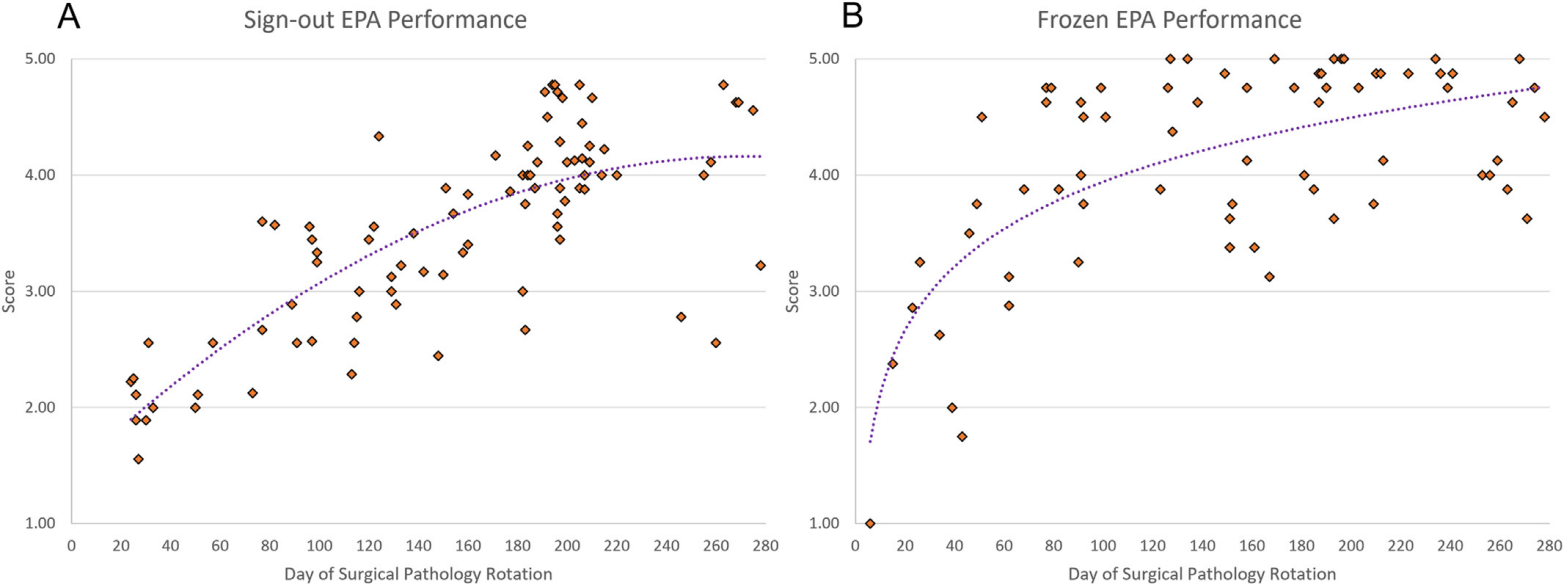
Preliminary EPA evaluation form responses for Sign-Out (A) and Frozens (B), showing the tracking of the progression of residents, demonstrating the average EPA form score across total workdays experienced in surgical pathology.

Cumulative form evaluations for Sign-out EPA and Frozen EPA were preliminary assessed over the one-year pilot program. A total of 88 Sign-out EPA evaluations and 70 Frozen EPA evaluation forms were collected during this time. Forms from residents across all time points in residency training were incorporated, providing a preliminary view of the academic development of pathology trainees (Fig. 3).

## Discussion

Despite CBME being acknowledged for decades as a valuable tool to prepare the trainee for unsupervised practice, there are few publications regarding implementation/experience with EPAs in United States pathology programs. In the current trainee assessment model, the ACGME milestones are evaluated semiannually by the program's clinical competency committee (CCC). Implementing EPAs with daily workplace-based assessment tools opens the channel for more frequent and on-site feedback, allowing the trainee to progress continuously towards independent, unsupervised practice. EPAs are an essential tool for program and rotation directors in determining the competence levels of each trainee.^21^

Recently-graduated pathologists frequently feel insecure in the first months of independent, unsupervised practice. Implementing EPAs in surgical pathology positively impacted other countries.^22^^,^^23^ In 2021, Gomes et al. reported implementing workplace-based assessment tools to assess residents' intraoperative consultations in a 5-year Anatomic Pathology Canadian program. Over a year, PGY-2 to PGY-5 residents' performances were assessed through their instrument, which contained 11 items. In this pilot, 13 residents were evaluated by 23 supervisors. Trainees and educators considered the EPA implementation to positively impact the learning process, emphasizing the shift towards a safe, confident, unsupervised practice. However, this pilot involved only intraoperative consultations and a 5-year AP-only Canadian curriculum.^23^

Implementing EPAs in surgical pathology can also assist the transition from medical school to pathology PGY-1. At the University College of Medical Sciences (New Delhi, India), Sharma et al. developed EPAs based on the resident's expectations for the end of PGY-1. In their surgical pathology rotation, their EPAs were developed focusing on the skills elected as “very important to acquire” in their training environment: grossing small specimens, grossing large specimens under supervision, proofreading reports, writing a good description of microscopic features, and making a biopsy diagnosis of nonmalignant endometrial aspirate.^21^

These two examples highlight that developing and implementing EPAs require keeping in mind locoregional and national needs, such as curriculum length, medical education culture, job opportunities, sign-out cycle style, the volume of surgical specimens, and intraoperative consultation. Gomes' and Sharma's perspectives appear promising in their countries, but they would only partially fill the specific needs of a surgical pathology curriculum in a US AP/CP 4-year residency program. Implementing EPAs is a complex task: each item should be carefully described to fit one's local and national expectations.

An appropriate EPA task should be written following Ten Cate's EPA principles (Table 5). Notably, each activity must be executable in a timeframe, observable and measurable in its process and outcomes, and directly evaluable. A well-written tool allows an adequate evaluation, in which performing well means being trusted to carry out that specific task unsupervised. Identifying such competence in a resident is valuable for the trainee and the whole learning environment: that individual can also be trusted as a teaching source for junior trainees.

**Table 5:** Specific features of EPAs (adapted from Ten Cate et al^2^).

1	Part of essential professional work.
2	Require adequate knowledge, skill, and attitude, generally acquired through training.
3	Lead to recognized output of professional labor.
4	Confined to qualified personnel.
5	Independently executable.
6	Executable within a time frame.
7	Observable and measurable in their process and outcome, leading to a conclusion “(“well done” vs “not well done”).
8	Should reflect one or more of the competencies to be acquired.

In our study, we developed 4 EPAs (Grossing, Frozens, Preview/Sign-out, and Professional Development), with progressive knowledge and skill assessments based on the outcome-based categories (Transition to Discipline, Foundations, Core, and Transition to Practice).^20^ Our EPAs were designed to: (1) follow Ten Cate's principles, (2) correlate individual EPAs with Miller's pyramid of clinical competency and ACGME supervision levels and benchmarks, (3) fulfill our internal deficiencies based on a retrospective assessment of nine years of our internal evaluations, and (4) prepare the trainees for the current US job market's expectations.^18^^,^^19^ We aimed to approach individuals in the whole spectrum of training, from the incoming PGY-1 to the graduating PGY-4.

Initial implementation of the EPAs became a mandated experience coinciding with the new academic year, in which it became a requirement for residents and faculty to become familiar with the basis of an EPA, the method of assessment, and the anticipated skills necessary to reach the outcomes-based progression. The standard of the surgical pathology rotation was altered so that each resident on service had access to evaluation forms. Additionally, evaluation forms were placed strategically in the frozens or sign-out areas, and completion of at least one EPA evaluation a week became a mandatory form of assessment to fulfill rotation expectations. Residents received real-time, face-to-face feedback and could implement daily actionable feedback to improve upon. Continued implementation of the EPA assessments benefited from the evaluation forms, in which faculty “EPA Champions” provided real-time feedback through evaluation on dedicated frozen or sign-out days.

The survey results about the ease of understanding, feasibility, and impact of the EPAs showed favorable responses from residents and faculty. Trainees viewed the EPAs positively, with ease of understanding, ease of implementation, and reflectiveness of feedback. Trainees also believed the forms gave them a better understanding of what they needed to improve, showcasing the power of the EPAs for professional development. Faculty members had similar responses and noted that the EPAs set good benchmarks for trainees and accurately reflected where a trainee was in their performance. These responses provide insight into how these EPAs are a powerful tool for feedback, education, and tracking of progression for trainees.

Still in active collection, the preliminary data visually represent the “learning curve” of pathology training pertaining to intraoperative consultations and case sign-out across the four years of residency training. The performance data demonstrate overall growth over time as expected and demonstrates instances of above or below-expectations performance compared to the expected average trend – a reflection of daily practice. Continuing evaluations are ongoing to highlight the real-time assessment's power and pinpoint individuals who may be “falling off the curve” for that specific EPA, in which interventions could be implemented promptly to bolster the trainee's performance, skills, and knowledge base. Further data collection and detailed analysis will be reported in a separate article in the near future.

Shifting a residency culture from the “timeframe” perspective to the “objective-goal perspective” can be challenging. However, utilizing a carefully selected group of EPAs that align with the ACGME milestones can assist the clinical competency committees by making it easier to evaluate the performance of an individual trainee on that specific rotation.^5^^,^^15^ Also, the EPAs should be a valuable tool for each resident to take ownership of their learning process and follow up on their progress.^17^, ^18^, ^19^^,^^24^

Although the EPAs are a promising tool in medical education, they should be carefully implemented. The trainees should be introduced to each specific group of tools in a timely manner. The reasons for shifting the evaluation method should also be clear. The evaluator, on the other hand, should be trained to perform a holistic evaluation that goes beyond the trainee's diagnostic skills: all facets of patient care should be taken into consideration, including quality of specimen care, efficiency, communication skills, and behavior in potentially hazardous situations. Finally, the evaluator should undertake specific training to mitigate intrinsic bias and provide a uniform grading methodology across multiple evaluators.

Our EPAs and evaluation methodology may be used as a reference for other surgical pathology programs; however, it is important to reinforce that they were developed taking into consideration all our internal and external variables, including but not limited to specific job market trends, sign-out cycle style, the volume of surgical specimens, and our intraoperative consultation schedule. Shifting surgical pathology education to an EPA-based assessment is challenging, and sharing experiences and results among the programs is desirable. Current barriers to implementation include form utilization, faculty uptake, and continued usage by trainees. We summarized our EPA conceptualization and implementation in the surgical pathology curriculum of a 4-year AP/CP US training, incorporating ACGME milestones and highlighting directly measurable outcomes.

Our institution has implemented our unique EPAs in the surgical pathology rotation for over a year. Trainees have been assessed utilizing the new EPA forms encompassing grossing, case previewing, sign-out preparedness, and frozen section competencies. An internal longitudinal assessment of our model transition is underway, including pre- and post-implementation surveys for trainees and evaluators. The future aims of the study are to assess trainee confidence, adequacy of feedback, and preparedness for independent practice after the implementation of the EPAs. Following the acquisition of additional data points, this information will be utilized to obtain an average growth curve (a graphical representation of the elusive “learning curve” of pathology) to map the development of the average resident and provide early insights into the professional development of each trainee. Additionally, we have developed an app-based software with digital access to the EPA forms, providing additional ease of use for the EPA form to complete and track trainee development.

## Conclusions

Implementing EPAs in surgical pathology education is feasible and has positively impacted residents' evaluation, including determining performance and setting improvement goals. With the current high utilization rate by dedicated faculty, we have successfully piloted our program in surgical pathology with preliminary data showing trends of trainee progression. In addition, our EPAs can be used as a reference for other surgical pathology programs, setting goals and guidelines for trainee performance in a competency-based performance setting.

## Declaration of competing interest

The authors declare that they have no known competing financial interests or personal relationships that could have appeared to influence the work reported in this paper.

## Funding

The article processing fee for this article was funded by an Open Access Award given by the Society of ‘67, which supports the mission of the 10.13039/100016205Association of Pathology Chairs to produce the next generation of outstanding investigators and educational scholars in the field of pathology. This award helps to promote the publication of high-quality original scholarship in *Academic Pathology* by authors at an early stage of academic development.
