# Supplementary material for: From principles to practice: Implementation of entrustable professional activities (EPAs) for surgical pathology residency education in a large academic hospital
Source: Acad Pathol. 2023 Nov 20;10(4):100097. doi: 10.1016/j.acpath.2023.100097 (PMC10679497; doi:10.1016/j.acpath.2023.100097)
Supplement: Multimedia component 1 [file mmc1.pdf]

Supplemental Data S1

Form 1A: Grossing Basics Checklist

**Resident**:	**Trainer**:
**Date**:	**Subspecialty Bench:** GSP / Breast / GI/GU / Gyn

The purpose of the assessment is to support resident learning and to assess how they performed TODAY. With that in mind, please use the scale below to evaluate each item, irrespective of the resident's stage/level of training – Please complete the form at the end of the day and also provide feedback to the resident.

SCORING SCALE			
	**1**	**I had to do it**	*Requires complete hands on guidance, did not do, or was not given the opportunity to do*
	**2**	**I had to talk them through**	*Able to perform the tasks but requires or demands constant direction*
	**3**	**I had to prompt them from time to time**	*Demonstrates some independence, but requires/demands intermittent direction*
	**4**	**I needed to be there just in case**	*Independent but unaware of risks, or not self-confident and still requires or demands supervision for safe practice*
	**5**	**I did not need to be there**	*Complete independence, understands risks, performs safely, independent grossing ready*

**Specimen type/procedure**							
			**Score**	**Score**	**Score**	**Score**	**Score**
1	Station cleanliness and organization	*Ensures station is clean, all supplies are available, and organized prior to beginning a new specimen*					
2	Specimen identification	*Ensures patient and specimen identity, and verifies all parts are accounted for*					
3	Use of PPE and safe sharps use	*Uses appropriate PPE, uses & disposes of sharps in a safe and appropriate manner*					
4	Obtaining relevant clinical, surgical and radiologic information	*Assesses required clinical, radiological, and prior pathological information, understands the intended surgical procedure and impact of pathological diagnosis*					
5	Specimen orientation	*Identifies key anatomical landmarks and/or uses surgical orientation cues to achieve correct orientation*					
6	Inking	*Demonstrates good inking technique to avoid over/under staining of tissue*					
7	Gross description	*Uses appropriate and accurate terminology of gross finding, including identification of lesions, sizes, distances, and margin status*					
8	Serially sectioning and sampling	*Cuts specimen into appropriately sized slices, follows grossing manual, submits appropriate sections and margins*					
9	Fixation	*Stores specimens in appropriate containers with sufficient fixative and in a safe location*					
10	Resident is able to safely and correctly gross this specimen type		Y / N	Y / N	Y / N	Y / N	Y / N

Signatures: ____________________ Resident ____________________ Trainer

Reproduced and adapted under Creative Commons License 4.0 from Gomes MM, Driman D, Park YS et al. Teaching and assessing intra-operative consultations in competency-based medical education: development of a workplace-based assessment instrument. Virchows Arch 2021;479:803–813.
https://link.springer.com/article/10.1007/s00428-021-03113-6

Form 1B: Grossing Log

Resident:

Trainer: put initial in boxes requiring direct supervision or performance	Observed (SP teach)	Direct supervision	Indirect supervision	4	5	6	7	8	9	10	11	12	13	14	15
**GSP**															
Skin ellipse															
Panniculectomy															
Skin: wide local excision															
Non-neoplastic amputations															
Adrenalectomy															
Thyroidectomy															
Parathyroidectomy															
Glossectomy															
Salivary gland resection															
Lymph node dissection															
Splenectomy															
Lobectomy															
Lung wedge															
Pleurectomy															
Cardiac valves															
Total laryngectomy															
Oral tumor resections															
Soft tissue/bone tumors															
**Breast**															
Reduction/Revision															
Gender affirming mastectomy, gynecomastia															
Excision w/o margins															
Excision w/ margins															
Mastectomy															
Neoadjuvant lumpectomy															
Neoadjuvant mastectomy															
**Gyn**															
Fibroids															
Prolapse/fibroid hysterectomy															
Benign ovary+/-fallopian tubes															
Hyperplasia/carcinoma/prophylactic hysterectomy															
Adnexal malignancy															
Cervical cone/LEEP															
Prophylactic BSO															
Vulvectomy															
Radical hysterectomy															
**GI**															
Benign bowel (diverticulosis, ischemia)															
Appendix															
Gallbladder															
Colectomy (benign/malignant)															
Whipple															
Low anterior resection															
Abdominoperineal resection															
Esophagectomy															
Endoscopic mucosal resection															
Gallbladder cancer															
Prophylactic gastrectomy															
**GU**															
Prostatectomy															
TURP/TURB															
Partial nephrectomy															
Orchiectomy															
Total cystectomy															
Radical nephrectomy															
**Perinatal**															
Singleton placenta															
Multigestational placentas															

Form 2: Intra-Operative Consultation Assessment

**Trainee**:	**Pathologist**:
**Date**:	**Service** (circle one): **Feinberg / Prentice**

The purpose of the assessment is to support resident learning and to assess how they performed TODAY. With that in mind, please use the scale below to evaluate each item, irrespective of the resident's stage/level of training – for the FIRST intra-operative (frozen section) consultation of the day. Please complete the form at the end of the procedure and also provide feedback to the resident.

SCORING SCALE			
	**1**	**I had to do it (*knows*)**	*Requires complete hands-on guidance, did not do, or was not given the opportunity to do (direct supervision)*
	**2**	**I had to talk them through (*knows how*)**	*Able to perform the tasks but requires or demands constant Direction (direct/indirect supervision)*
	**3**	**I had to prompt them from time to time (*shows how*)**	*Demonstrates some independence, but requires/demands intermittent direction (indirect supervision)*
	**4**	**I needed to be in the room just in case (*does*)**	*Independence but unaware of risks or not self-confident and still requires or demands supervision for safe practice (indirect supervision/oversight)*
	**5**	**I did not need to be there (*does*)**	*Completes tasks independently, understands the practice of pathology with knowledge and demonstrable behavior, practice ready, capable of providing oversight of junior residents (oversight)*

					**Score**
1	Pre-procedure plan	*Assesses required clinical/radiological and prior pathological information, understands the intended surgical procedure and impact of pathological diagnosis*			
2	Case preparation	*Ensures the frozen section room is ready for use (instruments/fixatives/reagents etc)*			
3	Surgery-pathology contract/handover	*Verifies clinical indication for intraoperative consultation, understands surgical approach and determines shared goals of care*			
4	Technical performance	*Efficiently performs steps (recording gross features, appropriate representative sections, orientation of tissue, handover to technologist etc) and preserves/prepares the specimen for final assessment*			
5	Diagnostic interpretation	*Locate and identify histological abnormalities, integrates clinical-radiological-pathological features, accounts for procedural limitations, provides a safe and accurate diagnosis in a timely fashion*			
6	Post-procedure plan	*Documents intraoperative consultation properly and handles/orients tissue appropriately for permanent pathological assessment*			
7	Efficiency and flow	*Economy of movement and flow; adequate handling of multiple specimens*			
8	Communication / Collaboration	*Professional and effective communication/collaboration with professional team (technologist, surgeon, circulating nurse, pathologist etc)*			
9	Resident is able to safely perform this procedure with (circle one) *(NB: This is a global assessment which does not require a score of 5 on all preceding categories.)*		(1) Direct supervision only	(2) Indirect supervision	(3) Oversight
10	Gave at least one specific aspect of procedure done well			YES	NO
11	Gave at least one specific suggestion for improvement			YES	NO

Signatures: ____________________ Pathologist ____________________ Resident

Reproduced and adapted under Creative Commons License 4.0 from Gomes MM, Driman D, Park YS et al. Teaching and assessing intra-operative consultations in competency-based medical education: development of a workplace-based assessment instrument. Virchows Arch 2021;479:803–813.
https://link.springer.com/article/10.1007/s00428-021-03113-6

Form 3: Sign Out Assessment

**Trainee**:	**Pathologist**:
**Date**:	**Service:** Breast / GI / GU / GYN / GSP / Other:

The purpose of the assessment is to support resident learning and to assess how they performed TODAY. With that in mind, please use the scale below to evaluate each item, irrespective of the resident's stage/level of training. Please complete the form at the end of sign out and also provide feedback to the resident.

		**(*Miller's pyramid of clinical competence*)**	*(ACGME supervision levels)*
SCORING SCALE	**1**	**I had to do it (knows)**	*Requires complete hands-on guidance, did not do, or was not given the opportunity to do (direct supervision)*
	**2**	**Some independence demonstrated (knows how)**	*Able to perform the tasks but requires or demands constant direction/prompting (direct/indirect supervision)*
	**3**	**Moderately independent (shows how)**	*Demonstrates some independence, but requires/demands intermittent direction/prompting (indirect supervision)*
	**4**	**Mostly independent (does)**	*Independence but unaware of issues or not self-confident, and still requires or demands minimal supervision/prompts for quality practice (indirect supervision/oversight)*
	**5**	**Fully independent (does)**	*Completes tasks without any guidance or intervention, understands the practice of pathology with knowledge and demonstrable behavior, practice ready, capable of providing oversight of junior residents (oversight)*

				**Score**
1	Obtaining relevant clinical, surgical and radiologic information	*Collected required clinical, radiological, and prior pathological information, understands the indications of the procedure, and the necessary synoptic summaries and forms needed in the report*		
2	Gross description	*The gross description is accurate, uses appropriate terminology, has the necessary measurements/parameters, submitted the minimal necessary blocks to answer the clinical questions, and is free of typographical errors*		
3	Case preview	*An accurate report is drafted before sign out. It's clear, organized, uses appropriate terminology, free of typographical errors, and answers the clinical questions*		
4	Staging	*If applicable, the report synoptic summary is completely filled out, with all the necessary parameters, margin status, and accurate staging*		
5	Diagnostic accuracy	*Able to correctly locate and identify critical, common and uncommon histologic findings, and understands the tissue changes.*		
6	Knowledge of disease	*Demonstrates understanding of normal histology, common and uncommon pathologies, and is knowledgeable of important clinical and demographic information, associations, immunohistochemical and molecular findings, and prognostic information*		
7	Differential diagnosis	*Able to formulate a reasonable differential diagnosis, with minimal ancillary testing needed (SS, IHC, ISH, cytogenetics, molecular) to resolve it*		
8	Systems based practice (SBP)	*Writes thoughtful and useful notes, considers costs of practice and outcomes for the patient in reporting, knowledgeable of billing, formulates ideas for improving care*		
9	Interpersonal and communication skills (ICS), Practice-based learning and improvement (PBLI)	*Demonstrates professional maturity, good communication with clinical and laboratory staff, takes initiative to request ancillary testing & resolve issues, troubleshoots, and takes ownership of cases*		
10	Resident is performing at the expected PGY level (circle one)		YES	NO
11	Gave at least one specific aspect of sign out done well		YES	NO
12	Gave at least one specific suggestion for improvement		YES	NO

Signatures: ______________________ Pathologist ______________________ Resident

Reproduced and adapted under Creative Commons License 4.0 from Gomes MM, Driman D, Park YS et al. Teaching and assessing intra-operative consultations in competency-based medical education: development of a workplace-based assessment instrument. Virchows Arch 2021;479:803–813.
https://link.springer.com/article/10.1007/s00428-021-03113-6

Form 4: Learning Plan

**Trainee**:	**Date**:	**Surg Path block #:**

1	**Goal**: *__S__pecific – __M__easurable – __A__ttainable – __R__elevant – __T__ime-bound*
2	**Goal**: *__S__pecific – __M__easurable – __A__ttainable – __R__elevant – __T__ime-bound*
3	**Goal**: *__S__pecific – __M__easurable – __A__ttainable – __R__elevant – __T__ime-bound*
	**Director comments/suggestions**

Signatures: __________________________ Director of Surgical Pathology __________________________ Resident

Form 5: Efficiency and Workflow Assessment

**Trainee**:	**Teaching Resident**:
**Date**:	

The purpose of the assessment is to analyze the workflow of the trainee with a focus on efficiency and economy of movement. With that in mind, please use the scale below to evaluate each item. Please complete the form during the observation of the resident during grossing and preview time.

SCORING SCALE			
	1	**Very inefficient**	*Requires major improvement or modification of current practices to improve efficiency. Would be significantly behind if working independently, or unable to do it*
	2	**Inefficient**	*Very slow or inefficient tasks. Able to do them, but requires or demands constant direction/prompting. Would finish tasks at an unreasonable time/very late if working independently.*
	3	**Somewhat efficient**	*Performs task at a slow but acceptable efficiency/workflow pace. Would finish later than an experienced resident but not at an unreasonable time if working independently*
	4	**Efficient**	*Performs tasks at a normal and acceptable efficiency/workflow pace. Completes tasks within a comfortable/normal time compared to peers.*
	5	**Optimal efficiency**	*Performs tasks with increased efficiency and workflow, does not waste time, completes tasks faster/more efficiently compared to peers*

**GROSSING**				**Score**
1	Specimen ID and collection	*Identifies and collects all parts pertaining to the case, ensures specimen identification*		
2	Chart review & data collection	*Obtains critical clinical, surgical, radiologic, pathologic and/or oncologic information necessary prior to beginning grossing*		
3	Orientation, measurements & inking	*Orients specimens quickly, obtains all necessary measurements, inks specimens efficiently and correctly*		
4	Sectioning, descriptions, sampling, and submission	*Sections specimens efficiently, quickly describes specimen findings, identifies needed sections, and samples these areas with economy of movement. Submits and documents cassettes without delay*		
**PREVIEWING**				**Score**
5	Chart review & data collection	*Obtains critical clinical, surgical, radiologic, pathologic and/or oncologic information necessary prior to beginning previewing the case*		
6	Slide review	*Previews slides with efficiency, quickly resolving and identifying histologic features, or quickly marking for question during sign out (does not engage in unnecessary stalling)*		
7	Report preparation	*Writes report with efficiency, knowing the necessary vocabulary and parameters that need to be included*		
8	Resident is able to gross and preview cases in an efficient manner, able to complete all necessary tasks within a reasonable time<br>*(NB: This is a global assessment which does not require a score of 5 on all preceding categories.)*		YES	NO
9	Give at least one specific aspect of procedure done well:			
10	Give at least one specific suggestion for improvement:			

Signatures: __________ Trainee __________ Teaching Resident

Form 6: Senior/Teaching Resident Evaluation

**Trainee**:	**Pathologist**:
**Date**:	

The purpose of the assessment is to evaluate the performance of the senior/teaching resident TODAY. With that in mind, please use the scale below to evaluate each item, irrespective of the resident's stage/level of training. Please complete the form at the end of the day and also provide feedback to the resident.

SCORING SCALE			
	**1**	**I had to do it**	*Requires complete hands on guidance, did not perform the task, or was not given the opportunity to do*
	**2**	**I had to talk them through**	*Able to perform the tasks but requires or demands constant direction/prompting*
	**3**	**I had to prompt them from time to time**	*Demonstrates some independence, but requires/demands intermittent direction/prompting*
	**4**	**I needed to be there just in case**	*Independent but unaware of issues or not self-confident, and still requires or demands supervision/prompts for quality practice*
	**5**	**I did not need to be there**	*Completes tasks independently, understands the practice of pathology with knowledge and demonstrable behavior, practice ready, capable of providing oversight of junior residents*

			**Score**	
1	Gross description review	*Ensures the gross description is accurate, is appropriately sampled, and addresses deficiencies and issues with the corresponding resident/PA*		
2	Pre-review preparation	*Asks relevant clinical, surgical, and radiologic information from the junior resident, and is able to articulate the importance of this information for case review*		
3	Case review	*Eloquent narration of the case during preview, accurately answers the junior resident questions regarding the findings or staging.*		
4	Post-review	*Effectively edits a concise, clear report, identifies gaps in necessary information, and is able to articulate the importance of each staging parameter*		
5	Trainee education	*Asks relevant, engaging, and educational questions, or engages in other educational activities, to assess and enhance the knowledge of the trainee*		
6	Ancillary testing	*Asks relevant questions pertaining ancillary testing (SS, IHC, ISH, cytogenetics, molecular), when and how to use them, and is able to explain the rationale for their use*		
7	Efficiency and flow	*Previews the case at a reasonable pace, efficient looking up information in the chart, and makes decisions within a reasonable time.*		
8	Feedback	*Provides positive, constructive, specific, and actionable feedback during sign out and at the end of the day; senior/teacher seeks feedback from trainee and faculty*		
9	Communication & Professionalism	*Maintains a positive learning environment, demonstrates patience, provides clear instruction and communication, and exemplifies professional behavior*		
10	Resident is performing as an effective teacher and role model (circle one)<br>*(NB: This is a global assessment which does not require a score of 5 on all preceding categories.)*		YES	NO
10	Give at least one specific aspect of the senior/teaching role done well:			
11	Give at least one specific suggestion for improvement:			

Signatures: ____________________ Pathologist ____________________ Resident

Reproduced and adapted under Creative Commons License 4.0 from Gomes MM, Driman D, Park YS et al. Teaching and assessing intra-operative consultations in competency-based medical education: development of a workplace-based assessment instrument. Virchows Arch 2021;479:803–813.
https://link.springer.com/article/10.1007/s00428-021-03113-6
